# Secondary myelodysplastic syndrome/acute myeloid leukaemia following mitoxantrone-based therapy for breast carcinoma

**DOI:** 10.1054/bjoc.2000.1196

**Published:** 2000-06-02

**Authors:** R Saso, S Kulkarni, P Mitchell, J Treleaven, G J Swansbury, J Mehta, R Powles, S Ashley, R Kuan, T Powles

**Affiliations:** Departments of Haematology, Medical Oncology, Cytogenetics, Statistics, The Royal Marsden Hospital, Downs Road, Sutton, SM2 5PT, UK; Austin & Repatriation Medical Centre, Victoria, West Heidelberg, Australia; Division of Transplantation Medicine, University of South Carolina, Columbia, SC, 29203, USA; Thames Cancer Registry, London, UK

**Keywords:** myelodysplastic syndrome, acute myeloid leukaemia, breast cancer, mitoxantrone

## Abstract

Of 1774 patients with breast cancer given mitoxantrone (MTZ) with methotrexate (*n* = 492) or with methotrexate and mitomycin C (*n* = 1282), nine developed MDS/AML after a median of 2.5 years. Median duration of survival from diagnosis of MDS/AML was 10 months and six patients died. The crude incidence of developing MDS/AML after MMM or MM chemotherapy was 15 per 100 000 patient years follow-up, while the actuarial risk was 1.1% and 1.6% at 5 and 10 years respectively. MTZ-based regimens carry a 10 × higher risk of subsequent MDS/AML compared to that seen in the general population. © 2000 Cancer Research Campaign


British Journal of Cancer (2000) **83**(1), 91–94
© 2000 Cancer Research Campaign
doi: 10.1054/ bjoc.2000.1196, available online at http://www.idealibrary.com on IDEAL®

### Secondary myelodysplastic syndrome/acute myeloid leukaemia following mitoxantrone-based therapy for breast carcinoma

**R Saso[1], S Kulkarni[2], P Mitchell[3], J Treleaven[1], GJ Swansbury[4], J Mehta[5], R Powles[2], S Ashley[6], A Kuan[7] and T Powles[2]**

Departments of [1]Haematology, [2]Medical Oncology, [4]Cytogenetics and [7]Statistics, The Royal Marsden Hospital, Downs Road, Sutton SM2 5PT, UK; [3]Austin & Repatriation Medical Centre, West Heidelberg, Victoria, Australia; [5]Division of Transplantation Medicine, University of South Carolina, Columbia SC 29203 USA, [6]Thames Cancer Registry, London, UK

**Summary** Of 1774 patients with breast cancer given mitoxantrone (MTZ) with methotrexate ($n$ = 492) or with methotrexate and mitomycin C ($n$ = 1282), nine developed MDS/AML after a median of 2.5 years. Median duration of survival from diagnosis of MDS/AML was 10 months and six patients died. The crude incidence of developing MDS/AML after MMM or MM chemotherapy was 15 per 100 000 patient years follow-up, while the actuarial risk was 1.1% and 1.6% at 5 and 10 years respectively. MTZ-based regimens carry a 10 × higher risk of subsequent MDS/AML compared to that seen in the general population. © 2000 Cancer Research Campaign

**Keywords:** myelodysplastic syndrome; acute myeloid leukaemia; breast cancer; mitoxantrone

Significant improvement in the management of breast carcinoma has been achieved over the last few decades, mostly because of the use of combinations of surgery, radiotherapy and chemoendocrine therapy (Powles et al, 1995; Fisher et al, 1997). Adjuvant and neoadjuvant chemotherapy is delivered with the intention of controlling micrometastases. The 20-year follow-up of the first combination chemotherapy regimen reported by Bonadonna et al (1995) involved cyclophosphamide, methotrexate and 5-fluorouracil. Various other combinations have been tried, and use of anthracyclines is reported to improve the results (Brincker et al, 1983); mitoxantrone has been tried in place of doxorubicin and may have fewer side-effects (Powles et al, 1991).

A number of chemotherapy drugs, especially alkylating agents and some anthracyclines used in breast cancer treatment, are associated with increased risk of secondary acute myeloid leukaemia (sAML) or myelodysplasia (MDS), (Pedersen-Bjergaard and Rowley, 1994). This analysis was undertaken in breast cancer patients to ascertain whether there is a similar risk of secondary AML/MDS associated with mitoxantrone.

### PATIENTS AND METHODS

The prospectively maintained database at the Royal Marsden Hospital was used to identify patients with breast carcinoma treated with mitoxantrone-based regimens in the period 1984–1995. Patients who developed leukaemia or MDS were identified in the Royal Marsden Hospital database and also by accessing data reported to the Thames Cancer Registry. Details from these sources were cross-checked and found to be consistent.

*Received 23 November 1999*
*Revised 23 February 2000*
*Accepted 23 February 2000*

*Correspondence to:* JG Treleaven

### Chemotherapy regimens

From 1984–1992, 1282 patients received MMM (mitoxantrone 7 mg $m^{-2}$ every 3 weeks, methotrexate 35 mg $m^{-2}$ every 3 weeks, mitomycin C 7 mg $m^{-2}$ every 6 weeks). From 1992 onwards mitomycin was excluded from the regimen since there was an increased incidence of haemolytic-uraemic syndrome (Montes et al, 1993), and the subsequent 492 patients were treated (in 1992–1995) with MM (methotrexate 35 mg $m^{-2}$ every 3 weeks, and mitoxantrone 11 mg $m^{-2}$ every 3 weeks). Patients also received local radiotherapy to the chest wall, and tamoxifen 20 mg daily. Table 1 summarizes treatments and characteristics of patients in the MMM and MM groups.

### Diagnosis of MDS/AML

MDS/AML was diagnosed using French-American-British (FAB) criteria (Bennett et al, 1982; 1985). Cytogenetic analysis was performed using conventional methods.

### Statistical methods

Patients were considered to be at risk of developing MDS/AML from the initial date of treatment with MMM or MM until the last follow-up or death. The actuarial incidence was calculated by the life-table method of Kaplan and Meier (1958). A comparison of patients treated with MMM and MM was carried out using the Logrank test. Survival after diagnosis of MDS/AML was also ascertained by the life-table method.

The expected numbers of acute myeloid leukaemias and myelodysplastic syndromes in an age/sex matched population were calculated by applying the age- and sex-specific figures for leukaemias in England and Wales (supplied by the Office of Population Census and Surveys) assuming a Poisson distribution to the person-years at risk. The age-specific incidence figures for MDS were obtained from the data published by Williamson et al (1994). The relative risk of developing AML/MDS was calculated

**Table 1** Patients treated with MM/MMM

| | MMM | MM |
|---|---|---|
| Number of patients | 1282 | 492 |
| Male/Female | 5/1277 | 1/491 |
| Age (median (range)) | 54 (20–90) | 54 (27–83) |
| Local RT | 366 | 319 |
| No RT | 214 | 84 |
| RT (no information)[a] | 702 | 89 |
| Number of courses (median (range)) | 6 (1–15) | 6 (1–11) |

[a] RT (local radiotherapy) data not available before April 1988

using the standardized incidence ratio, and confidence intervals are exact (Breslow and Day, 1987).

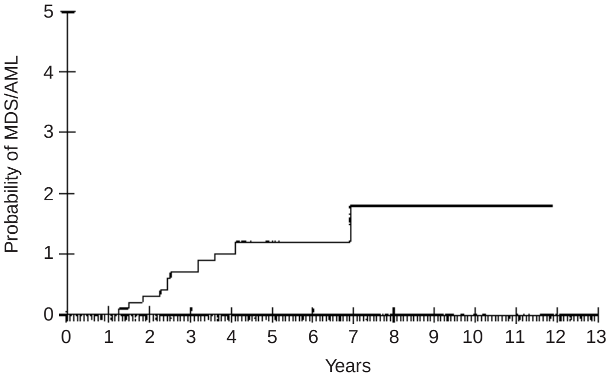

**Figure 1** Incidence of MDS/AML after MZT-based chemotherapy.

### RESULTS

In the period 1984–1995 1774 patients received MMM chemotherapy ($n$ = 1282, median age 54 years, range 20–90) or MM ($n$ = 492, median age 54 years, range 27–83) in adjuvant or neoadjuvant setting. Median follow-up was 5 years.

Five patients developed AML and four developed MDS after treatment with MTZ-based chemotherapy, giving an incidence of 15 per 100 000 years follow-up. The median age was 60 years (range 45–68). Details of the cases of MDS and AML are shown in Table 2. On average, MDS/AML developed 2.5 years (range 1.3–7) following MTZ-based chemotherapy (Table 2). There was no difference in incidence between prior MM or MMM chemotherapy (3/492 versus 6/1282; $P$ = 0.9). The risk of developing MDS/AML at 5 and 10 years was calculated as 1.1% (95% CI: 0.6–2.2%) and 1.6% (95% CI: 0.7–3.6%) respectively (Figure 1). The relative risk of developing MDS/AML was 10.1 times greater than in an age- and sex-matched normal population (95% CI: 3.5–16.7). For MDS alone the risk was 8.6 times greater, and for leukaemia alone it was 14.1 times greater than in the normal population (Table 3).

Women with AML/MDS had received the following total drug doses: Mitoxantrone 80 mg (range 60–162 mg), methotrexate 310 mg (range 265–400 mg), in six cases with mitomycin 45 mg (range 39–51 mg). In addition to MTZ-based chemotherapy, all patients had surgery and additional chest wall irradiation, while five patients in this group had adjuvant therapy with tamoxifen. An abnormal cytogenetic clone was present in eight cases, while one patient had no clonal abnormality. Five individuals had abnormalities involving chromosomes 5 and/or 7, and three had

**Table 2** Details of secondary MDS/AML patients

| Case | Age (years) | Diagnosis | Ca. Breast T/T[a] | Regimen | Time to AML/MDS (years) | Total chemotherapy doses[d] | | | Cytogenetics | Outcome (months) |
|---|---|---|---|---|---|---|---|---|---|---|
| | | | | | | Mitoxantrone | Methotrexate | Mitomycin | | |
| A | 64 | AML M7 | S, CT+RT, Tamox | MMM | 3.6 | 68 mg (46.9) | 270 mg (186.2) | 39 mg (26.9) | 45, XX, –7 | Dead 2 |
| B | 50 | AML M1 | S, CT+RT | MMM | 4.2 | 60 mg (37.5) | 300 mg (187.5) | 45 mg (28.1) | 47, XX, +mar | Dead 1 |
| C | 71 | MDS/RA | CT, S, CT+RT, Tamox | MMM | 3.2 | 79 mg (45.1) | 310 mg (177.1) | 45 mg (25.7) | 46, XX | Dead 10 |
| D | 46 | AML M2 | CT, S, CT+RT | MM | 1.3 | 162 mg (93.1) | 400 mg (229.9) | nil | 46, XX, t(8;21)(q22;q22) | In CR1 60 |
| E | 75 | MDS Hypoplas | HAD[c], S, HAD+RT | MM FEC[b] | 2.5 | 96 mg (59.6) | 320 mg (198.8) | nil | 46, XX, del(1)(p32p34), del(5)(q13q33), der(7)t(7;12)(q3;q13), der(12)t(7;12)(q22;q13) | Dead 17 |
| F | 58 | AML M3 | CT, S, CT, RT, Tamox | MM | 1.8 | 126 mg (70) | 300 mg (166.7) | nil | 46, XX, t(1;2)(p22;q21), t(15;17)(q22;q21) | In CR1 18 |
| G | 66 | MDS RARS | CT, S, CT, RT, Tamox | MMM | 2.4 | 77 mg (38.5) | 265 mg (132.5) | 50 mg (25) | 45, XX, del(3)(p12),–7, –9 add(17)(p1), –8, +mars/ 45, XX, idem, del(5)(q3) | Dead 8 |
| H | 62 | MDS | S, RT, Tamox | MMM | 7 | 93 mg (51.7) | 320 mg (177.8) | 51 mg (28.3) | 46, XX, –7, +mar | Alive 12 |
| I | 77 | AML M2 | S, RT, Tamox, VAC/VEC | MMM | 2.4 | 80 mg (53.3) | 400 mng (260.6) | 40 mg (26.6) | 45, XX, add(5)(q15), der(11) add(11)(p15)add(11)(q23), –18, del(20)(q13) | Dead 27days |

[a] S = surgery, CT = chemotherapy, RT = radiotherapy; [b]FEC-5FU, epirubicin, cyclophosphamide, VAC/VEC = vincristine, adriamycin (epirubicin), cyclophosphamide, Tamox = tamoxifen; [c]HAD-hydroxyandrostenedione; [d]Total dose in mg, mg $m^{-2}$ in parentheses

 © 2000 Cancer Research Campaign

**Table 3** Relative risk (RR) of developing MDS/AML compared to an age- and sex-matched population

| | No. of cases | RR | 95% CI | Significance |
|---|---|---|---|---|
| MDS/AML | 9 | 10.1 | 3.5–16.7 | $P < 0.01$ |
| MDS | 4 | 8.6 | 0.2–17.0 | $P < 0.1$ |
| AML | 5 | 14.1 | 2.8–25.4 | $P < 0.05$ |

complex abnormalities. Other chromosome abnormalities included t(8;21), t(15;17) with t(1;2), and one patient had an unidentified additional abnormality (Table 2).

Patient outcomes are shown in Table 2. All five patients with AML were treated with chemotherapy. Two are alive, and three died of chemotherapy-related problems. Four patients with MDS were over 60 years-of-age and were managed with supportive care alone; three have died. In one patient the MDS evolved into AML.

### DISCUSSION

There has been significant improvement in survival and cure expectancy for patients with breast cancer due to combined modality treatment using surgery, chemotherapy and radiation. Selection of the optimum form of combination chemotherapy for breast cancer should take into account associated long-term risks. The CMF regimen is a standard regimen for adjuvant treatment and its long-term benefit has been established. However, there is a potential risk of secondary leukaemia associated with most of these drugs (Henderson et al, 1990).

Aul et al (1992) have shown that the risk of developing MDS/AML increases with age; the average annual incidence in all age-groups in the period 1986–1990 was 4.11 per 100 000 per year, with a considerable rise of incidence in the older population. The average incidence in the population > 70 years-of-age was 22.81 per 100 000 per year (33.88 for men and 18.02 for women), while the incidence of AML is 6.7 per 100 000 per year. Another study has confirmed an increased incidence of MDS in older patients (Williamson et al, 1994).

We assessed the incidence of MDS and AML occurring in a cohort of patients with breast cancer treated with mitoxantrone-containing regimens. The current report is one of the largest analyzing risk of secondary acute myeloid leukaemia after mitoxantrone in breast cancer patients. With a median follow-up of 5 years, there were nine cases of AML/MDS among 1774 patients giving a crude incidence of 15 per 100 000 years of follow-up.

Bonadonna et al (1993) noticed a cumulative risk of 0.23% +/– 0.15% at 15 years, and a relative risk 2.3 × greater compared to the normal population after a median follow-up of 12 years in patients receiving CMF. However, the Eastern Cooperative Oncology Group (ECOG) found a crude incidence rate of 26 per 100 000 years of follow-up for a secondary haematological malignancy after treatment with standard adjuvant chemotherapy, which with standard dose cyclophosphamide was not much higher than that seen in the general population (Tallman et al, 1995).

To improve tumour response rates and prolong survival, anthracycline-based regimens have been introduced, with results at least as good as those seen with CMF. However, the risk of secondary AML with doxorubicin-containing regimens has been found to be 1.5% (0.7–2.9%) at 10 years (Diamandidou et al, 1996) and the risk was significantly higher after chemoradiotherapy than after chemotherapy alone (10 year actuarial risk of 2.5% vs 0.5%). Mitoxantrone may be less toxic and has also been used more recently in combination chemotherapy for breast cancer (Powles et al, 1991).

To our knowledge there is no association between mitomycin and methotrexate and hematological malignancies (Rustin et al, 1996), and there are few reports concerning a possible leukaemogenic effect of mitoxantrone (Philpott et al, 1993; Mitchell et al, 1996). Therapy-related or secondary leukaemias often show cytogenetic abnormalities, and there are characteristic differences which may be specific for particular chemotherapy agents; alkylating agents are associated with abnormalities of chromosomes 5 and 7 while topoisomerase-II inhibitors are associated with chromosome 11 abnormalities. (Ellis et al 1993; Smith et al, 1996).

In our study, 67% of patients had predominantly unbalanced chromosome aberrations that are associated with prior radiotherapy and/or alkylating agent treatment, but not with mitoxantrone therapy. However, all the patients with MDS/AML had also received radiotherapy and hence it is possible that in our patients the risk of MDS/AML was more likely to be associated with the combination of chemotherapy and radiotherapy (Johansson et al, 1996).

Two patients in this series who had balanced translocations had a family history of cancer. One had a sister with brain cancer, while the other had a mother with lung cancer and a sister with breast cancer. Because of their family backgrounds, these patients may be in a high-risk category and more prone to developing a secondary malignancy; MDS/AML in this situation is more likely to represent a second primary rather than a secondary haematological malignancy (Thirman and Larson, 1996). An increased family incidence of haematological malignancies in these cases may also indicate a role for oncogenes and tumour suppressor genes in the development of MDS/AML (Skuse and Ludlow, 1995).

### CONCLUSION

Our study has shown an increased incidence of MDS and AML after MTZ-based chemotherapy, compared to that seen in the general population. However, it is difficult to estimate how much of this is due to the MTZ-based chemotherapy rather than to the population being at higher risk because of family history, age or additional treatment with chemo/radiotherapy.

### ACKNOWLEDGEMENTS

We are grateful to Ms Carol Brooker of Queen's Hospital, Croydon, for cytogenetic data of case H.

### REFERENCES

Aul C, Gattermann N and Schneider W (1992) Age-related incidence and other epidemiological aspects of myelodysplastic syndromes. *Br J Haematol* **82**: 358–367

Bennett JM, Catovsky D, Daniel MT, Flandrin G, Gahon DA, Gralnick HR and Sultan C (1982) FAB Cooperative Group. Proposals for the classification of myelodysplastic syndromes. *Br J Haematol* **51**: 189–199

Bennett JM, Catovsky D, Daniel MT, Flandrin G, Galton D, Gralnick HR and Sultan C (1985) Proposed revised criteria for the classification of acute myeloid leukemia. *Ann Intern Med* **103**: 620–625

© 2000 Cancer Research Campaign 

Bonadonna G, Valagussa P, Moliterni A, Zambetti M and Terenziani M (1993) Risk of acute leukemia and other malignances following CMF-based adjuvant chemotherapy for breast cancer. *Proceedings of the American Society of Clinical Oncologists* **12**: 61 (abstr 45)

Bonadonna G, Valagussa P, Moliterni A, Zambetti M and Brambilla C (1995) Adjuvant cyclophosphamide, methotrexate, and fluorouracil in node-positive breast cancer. The results of 20 years of follow-up. *N Engl J Med* **332**: 901–906

Breslow NE and Day NE (1987) The design and analysis of cohort studies. In *Statistical methods in Cancer Research*, Vol 2. pp 1–406. IARC Scientific Publications: Lyon

Brincker H, Rose C, Maase H and Dombernowsky P (1983) A randomized study of CAF-TAM (Tamoxifen) versus CMF + TAM in metastatic breast cancer. *Proceedings of the American Society of Clinical Oncologists* **3**: 113

Diamandidou E, Buzdar AU, Smith TL, Frye D, Witjaksono M and Hortobagyi GN (1996) Treatment-related leukemia in breast cancer patients treated with fluorouracil-doxorubicin-cyclophosphamide combination adjuvant chemotherapy: The University of Texas MD Anderson Cancer Center Experience. *J Clin Oncol* **14**: 2722–2730

Ellis M, Ravid M and Lishner M (1993) A comparative analysis of alkylating agent and epipodophyllotoxin-related leukemias. *Leuk Lymphoma* **11**: 9–13

Fisher B, Dignam J, Wolmark N, DeCillis A, Emir B, Wickerham DL, Bryant J, Dimitrov NV, Abramson N, Atkins JN, Shibata H, Deschenes L and Margolese RG (1997) Tamoxifen and chemotherapy for lymph node-negative, estrogen receptor-positive breast cancer. *J Natl Cancer Inst* **89**: 1673–1682

Henderson IC, Garber JE, Breitmeyer JB and Hayesris JR (1990) Comprehensive management of disseminated breast cancer. *Cancer* **66**: 1439–1448

Johansson B, Mertens F and Mitelman F (1996) Secondary neoplasia-associated chromosomal abnormalities-Balanced rearrangements vs genomic imbalances? *Genes Chromosomes Cancer* **16**: 155–163

Kaplan EL and Meier P (1958) Nonparametric estimation from incomplete observations. *American Statistical Association Journal* **53**: 457–481

Mitchell PL, Treleaven JG, Swansbury GJ, Powles RL, Ashley S and Powles T (1996) Secondary acute myeloid leukaemia (AML) and myelodysplasia (MDS) following mitoxantrone given as adjuvant therapy for breast cancer. *Proceedings of the American Society of Clinical Oncologists* **12**: 127

Montes A, Powles TJ, O'Brien ME, Ashley SE, Luckit J and Treleaven J (1993) A toxic interaction betwen mitomycin C and tamoxifen causing the haemolytic uraemic syndrome. *Eur J Cancer* **29A**: 1854–1857

Pedersen-Bjergaard J and Rowley J (1994) The balanced and unbalanced chromosome aberrations of acute myeloid leukemia may develop in different ways and may contribute differently to malignant transformation. *Blood* **83**: 2780–2786

Philpott NJ, Bevan DH and Gordon-Smith EC (1993) Secondary leukaemia after MMM combined modality therapy for breast carcinoma. *Lancet* **341**: 1289

Powles TJ, Jones AL and Judson IR, et al. (1991) A randomised trial comparing combination chemotherapy using mitomycin C, mitoxantrone and methotrexate (3M) with vincristine, anthracycline and cyclophosphamide (VAC) in advanced breast cancer. *Br J Cancer* **64**: 406–410

Powles TJ, Hickish TF, Makris A, Ashley SE, O'Brien M, Tidy VA, Casey S, Nash A, Sacks N, Cosgrove D, MacVicar D, Fernando I and Ford HT (1995) Randomized trial of chemoendocrine therapy started before and after surgery for treatment of primary breast cancer. *J Clin Oncol* **13**: 547–552

Rustin GJS, Newlands ES, Lutz JM, Holden L, Bagshawe KD, Hiscox JG, Foscett M, Fuller S and Short D (1996) Combination but not single-agent methotrexate chemotherapy for gestational trophoblastic tumors increases the incidence of second tumors. *J Clin Oncol* **14**: 2769–2773

Skuse GR and Ludlow JW (1995) Tumour suppressor genes in disease and therapy. *Lancet* **345**: 902–906

Smith MA, McCaffrey RP and Karp JE (1996) The secondary leukemias: Challenges and research directions. *J Natl Cancer Inst* **88**: 407–418

Tallman MS, Gray R, Bennett JM, Variakojis D, Robert N, Wood WC, Rowe JM and Wiernik PH (1995) Leukemogenic potential of adjuvant chemotherapy for early stage breast cancer: The Eastern Cooperative oncology group experience. *J Clin Oncol* **13**: 1557–1563

Thirman MJ and Larson RA (1996) Therapy related myeloid leukemia. *Hematol Oncol Clin North Am* **10**: 293–320

Williamson PJ, Kruger AR, Reynolds PJ, Hamblin TJ and Oscier DG (1994) Establishing the incidence of myelodysplastic syndrome. *Br J Haematol* **87**: 743–745

*© 2000 Cancer Research Campaign*
